# Supplementary material for: Spray Flame Synthesis (SFS) of Lithium Lanthanum Zirconate (LLZO) Solid Electrolyte
Source: Materials (Basel). 2021 Jun 22;14(13):3472. doi: 10.3390/ma14133472 (PMC8269458; doi:10.3390/ma14133472)
Supplement: Supplementary file 1 [file materials-14-03472-s001.zip › materials-1213650-supplementary.pdf]

materials

Supplementary Material

Spray Flame Synthesis (SFS) of Lithium Lanthanum Zirconate (LLZO) Solid Electrolyte

Md Yusuf Ali [1], Hans Orthner [1] and Hartmut Wiggers [1,2,*]

[1] IVG, Institute for Combustion and Gas Dynamics—Reactive Fluids, University of Duisburg-Essen, 47057 Duisburg, Germany; yusuf.ali@uni-due.de (M.Y.A.); hans.orthner@uni-due.de (H.O.)
[2] CENIDE, Center for Nanointegration Duisburg-Essen, 47057 Duisburg, Germany
* Correspondence: hartmut.wiggers@uni-due.de

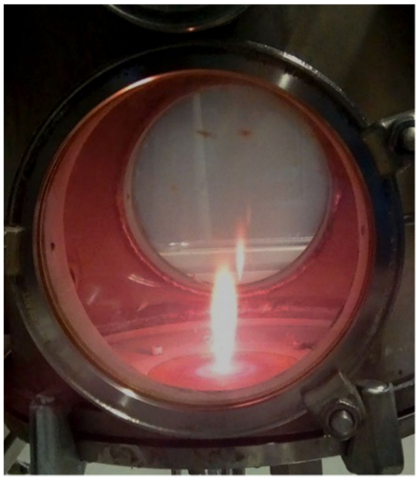

Figure S1. Photo of the spray flame taken during the synthesis of LLZO. The red coloration is due to the flame coloration by the lithium.

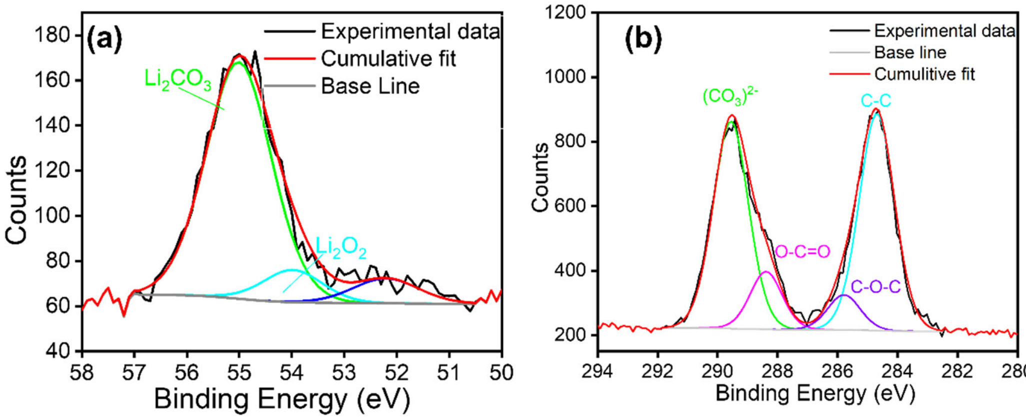

Figure S2. Results of XPS measurements (Li 1s (**a**) and C 1s (**b**)) of the material produced from Sol$_{LLZO1}$. The particles' surface contains mainly lithium species, which was – based on the C 1s data—identified as lithium carbonate.

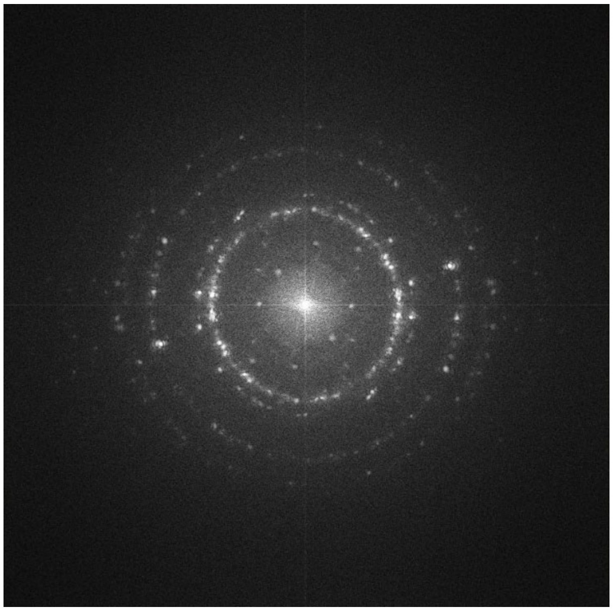

Figure S3. Electron diffraction pattern of sample LLZO2.

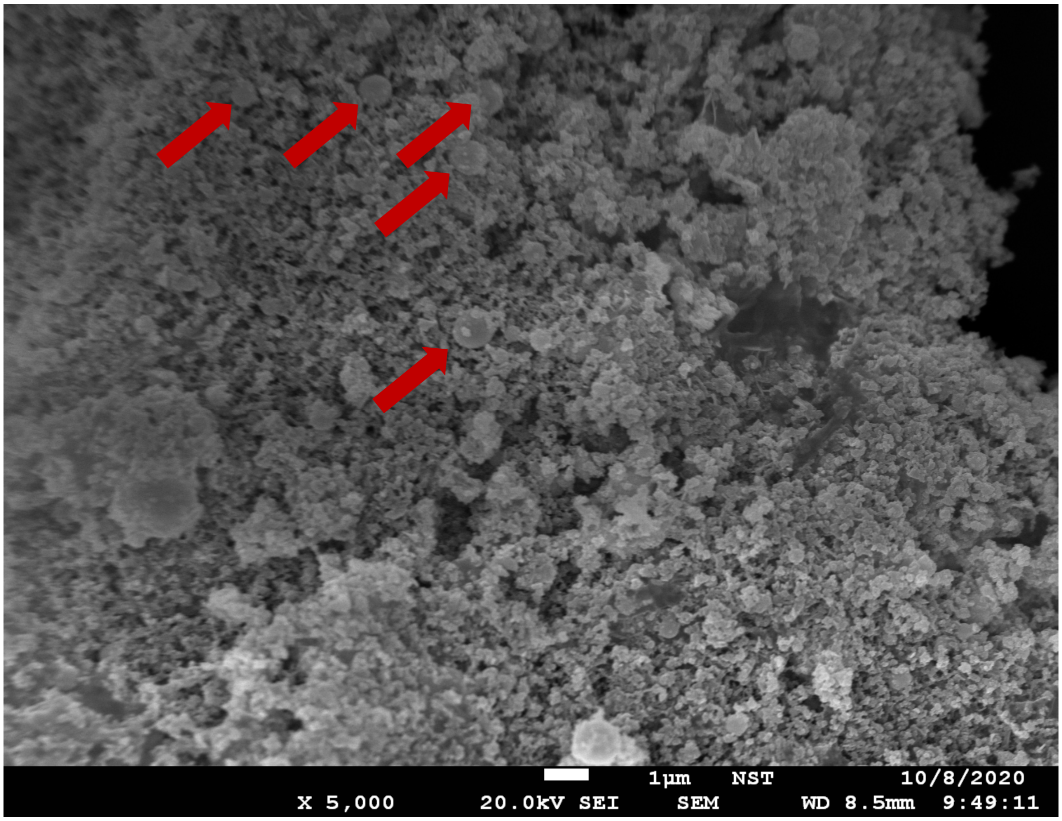

Copyright: © 2021 by the authors. Licensee MDPI, Basel, Switzerland. This article is an open access article distributed under the terms and conditions of the Creative Commons Attribution (CC BY) license (http://creativecommons.org/licenses/by/4.0/).

Figure S4. FESEM image of as-synthesized powder from Sol_{LLZO1}. In addition to the expected nanopowder, spherical particles of a few hundred nanometers can be seen in isolated cases (see red arrows), which are not formed by a gas-to-particle process but by a droplet-to-particle formation process [1].

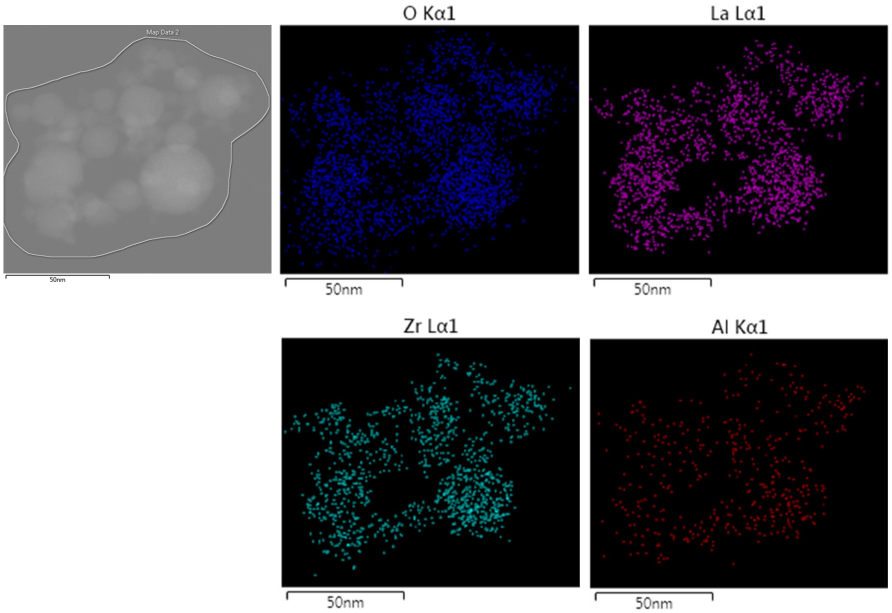

Figure S5. Elemental mapping of as-synthesized LZO indicating a homogeneous distribution of the metals within the particles.

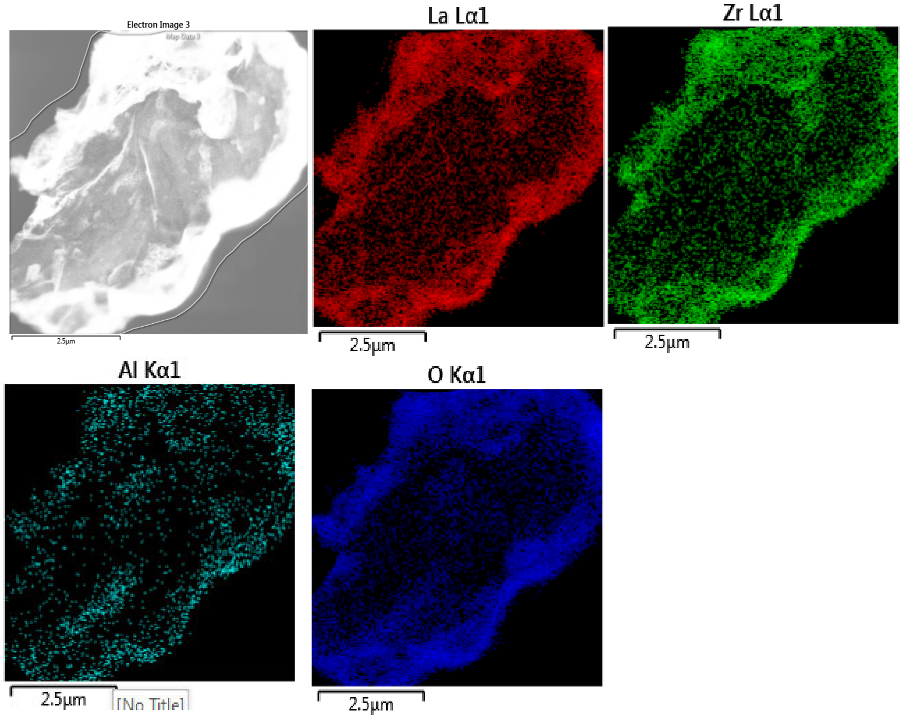

Figure S6. Elemental mapping of as-synthesized LLZO1, again indicating a homogeneous distribution of La, Zr, and Al.

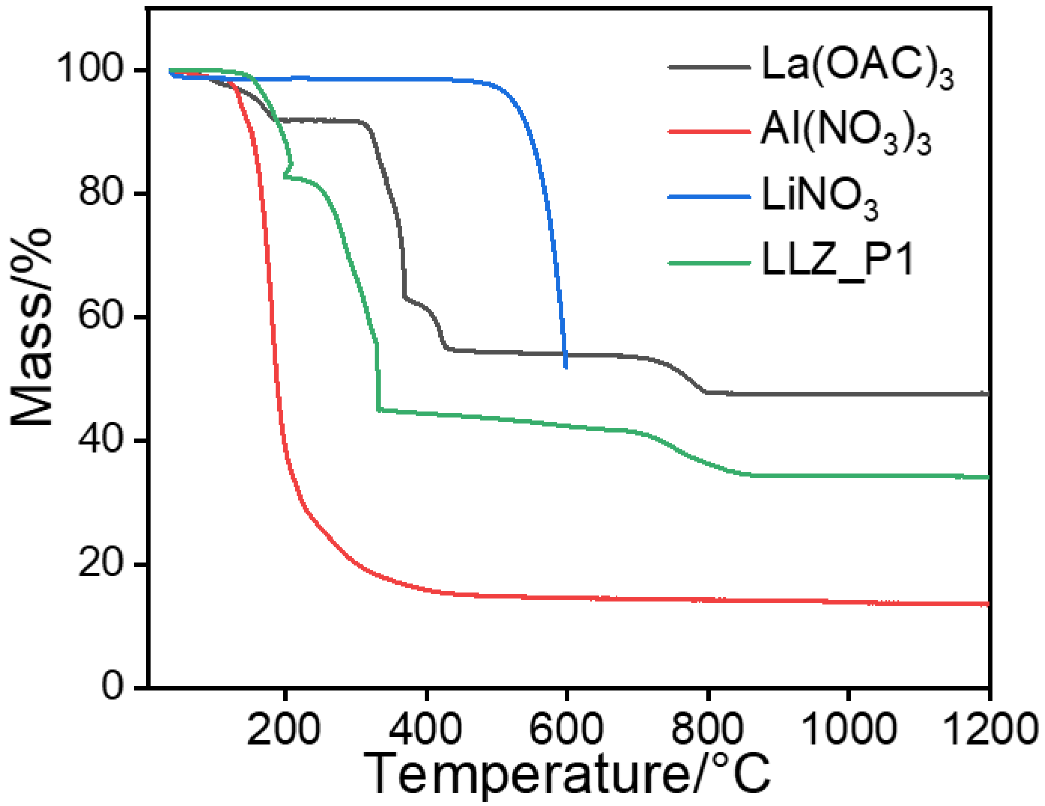

Figure S7. TGA of precursors used and a dried precursor solution LLZ_P1 (LLZO2 precursor solution was evaporated, and the resulting paste was dried). The powders were pressed into pellets before being measured by TGA. Although of the nitrates $LiNO_3$ has the highest temperature stability, all nitrates decompose at 600 °C. The lanthanum precursor decomposes in several stages, with the most thermally stable compound probably being $La_2O_2CO_3$ as identified in previous work [2].

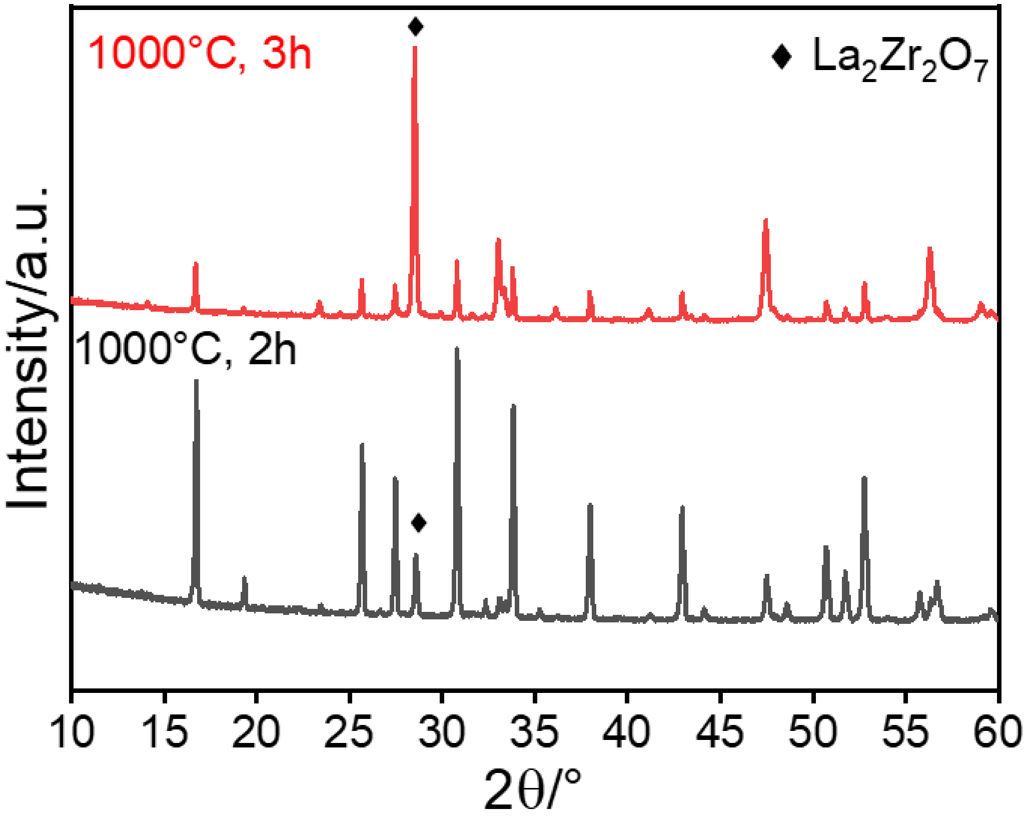

Figure S8. XRD of heated sample of LLZO2 at 1000°C, for 2h (black) and 3h (red). .

References:

1. Gurav, A.; Kodas, T.; Pluym, T.; Xiong, Y. Aerosol Processing of Materials. *Aerosol Sci. Technol.* **1993**, *19*, 411–452, doi:10.1080/02786829308959650.
2. Alkan, B.; Cychy, S.; Varhade, S.; Muhler, M.; Schulz, C.; Schuhmann, W.; Wiggers, H.; Andronescu, C. Spray-Flame-Synthesized LaCo 1− x Fe x O 3 Perovskite Nanoparticles as Electrocatalysts for Water and Ethanol Oxidation. *ChemElectroChem* **2019**, *6*, 4266–4274, doi:10.1002/celc.201900168.
